# Impact of Air Pollution and Weather on Dry Eye

**DOI:** 10.3390/jcm9113740

**Published:** 2020-11-20

**Authors:** Jordan T. Mandell, Maria Idarraga, Naresh Kumar, Anat Galor

**Affiliations:** 1 Miller School of Medicine, University of Miami, Miami, FL 33136, USA; j.mandell@med.miami.edu; 2 Bascom Palmer Eye Institute, University of Miami, Miami, FL 33136, USA; mai47@miami.edu; 3 Epidemiology and Public Health, University of Miami, Miami, FL 33136, USA; nkumar@med.miami.edu; 4 Ophthalmology, Miami Veterans Affairs (VA) Medical Center, Miami, FL 33136, USA

**Keywords:** dry eye, air quality, outdoor air pollution, indoor air pollution

## Abstract

Air pollution has broad effects on human health involving many organ systems. The ocular surface is an excellent model with which to study the effects of air pollution on human health as it is in constant contact with the environment, and it is directly accessible, facilitating disease monitoring. Effects of air pollutants on the ocular surface typically manifest as dry eye (DE) symptoms and signs. In this review, we break down air pollution into particulate matter (organic and inorganic) and gaseous compounds and summarize the literature regarding effects of various exposures on DE. Additionally, we examine the effects of weather (relative humidity, temperature) on DE symptoms and signs. To do so, we conducted a PubMed search using key terms to summarize the existing literature on the effects of air pollution and weather on DE. While we tried to focus on the effect of specific exposures on specific aspects of DE, environmental conditions are often studied concomitantly, and thus, there are unavoidable interactions between our variables of interest. Overall, we found that air pollution and weather conditions have differential adverse effects on DE symptoms and signs. We discuss these findings and potential mitigation strategies, such as air purifiers, air humidifiers, and plants, that may be instituted as treatments at an individual level to address environmental contributors to DE.

## 1. Introduction

Air pollution is becoming an increasing global concern, especially outdoor pollution in low-income countries and indoor air pollution in high income countries [1]. Most studies have focused on the link between air pollution and cardiopulmonary diseases, such as asthma, chronic obstructive pulmonary disease (COPD), lung cancer, heart disease, and stroke [2,3]. Emerging literature further suggests that air pollution affects other systems as well, such as the reproductive system, neurological system, and the eye. For example, studies have linked air pollution to male and female infertility [4,5], psychological burden [6], and neurodegeneration [7].

Air pollution includes a complex mixture of particulate matter (PM) and harmful gases. Organ specific PM effects are dictated by the size, morphology, and composition of particles. The two well-established PM sizes PM_2.5_ (≤2.5 µm) and PM_10_ (≤10 µm) have different target organs. For example, as PM_2.5_ passes through the pulmonary system, some of it is deposited in the lower airways and the rest reaches alveoli and therefore the circulatory system. Thus, it affects the cardiopulmonary systems. PM_10_, on the other hand, does not reach the alveoli, and its effects are restricted to the pulmonary system. If particles are in ultrafine ranges (PM_0.1_ (≤0.1 µm)), they can further penetrate endothelial layers. PM is further categorized by composition into organic and inorganic matter. Organic PM includes airborne bioaerosols, which are particles from microbes, plants, and animals that are suspended in the air. All other airborne particles fall under the category of inorganic PM, which can include dust (silica), calcium and heavy metal particles such as lead. Gaseous air pollutants include reactive gases, legislated by the US Environmental Protection Agency (EPA), as carbon monoxide (CO), ozone (O_3_), nitrogen dioxide (NO_2_), sulfur dioxide (SO_2_), and volatile organic compounds (VOCs). The pathologic mechanisms of air pollutants that underlie disease likely vary by air pollution type, but shared mechanisms include induction of oxidative stress and inflammation with subsequent nerve, vascular, and DNA damage [8,9].

**Figure 1:**
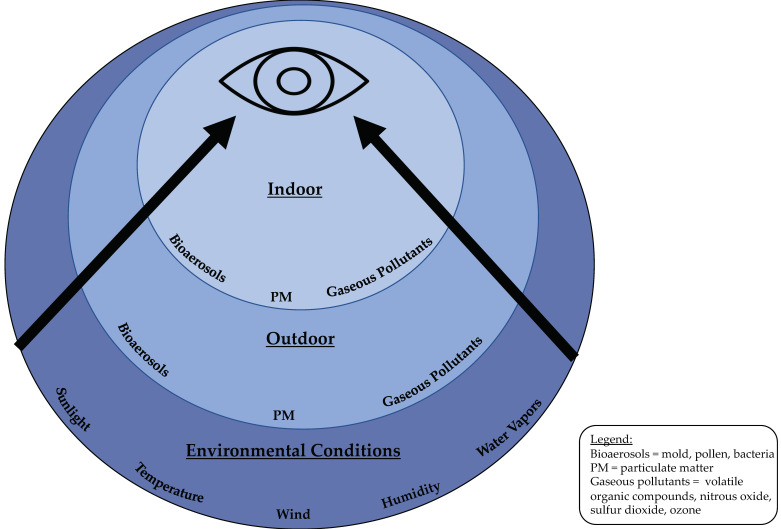
Concomitant environmental conditions affecting the ocular surface.

Until recently, most studies have focused on the effects of outdoor air pollution on human health [10]. However, indoor air pollution is an important contributor to human health, especially given that individuals spend an estimated 80–90% of their day in indoors [11]. Moreover, indoor and outdoor environments are not mutually exclusive as bioaerosols, gases, and particulate matter exchange between indoor and outdoor environment through windows, doors, and other openings, with meteorological conditions present in both settings (Figure 1). However, sources of indoor and outdoor air pollutants are quite different and rate of air exchange between indoor and outdoor environment impacts the levels and types of air pollutants found indoors. Understanding the unique consequences of a particular air pollutant on the body is important given that indoor air is potentially modifiable through simple interventions.

The ocular surface is an excellent model with which to study the effect of various air pollutants on human health as it is in constantly in direct contact with the environment and airborne matter and it can be non-invasively accessible, facilitating disease monitoring. The ocular surface includes the corneal and conjunctival epithelial layer, nerves, and tear lake [12] and serves as a barrier to chemicals, microbes, water, and other substances to protect the eye. A healthy eye should maintain a moist surface in various conditions thereby allowing individuals to remain asymptomatic throughout various physical and biological aggressions. However, air pollutants and weather conditions can compromise tear film and ocular surface health and affect the eye’s ability to lubricate and protect itself. In addition, adverse environmental conditions can activate corneal nerves, leading the dry eye symptoms that include sensations of dryness, aching, tenderness, and burning, to name a few [13]. The ocular surface is easily examined with a slit lamp and disease metrics quantified such as tear break up time (TBUT, lower scores more abnormal), corneal epithelial cell disruption (e.g., staining, higher scores more abnormal), and tear production via Schirmer strips (lower scores more abnormal). While with other organs, biospecimen collection can be challenging, in the eye tear collection is easy and non-invasive. For example, impression cytology can be performed to examine the superficial layers of the ocular epithelium.

The goal of this review is to assess the state of knowledge on the effects of air pollution (PM and gases) and weather on symptoms and signs of dry eye (DE) and to examine whether severity and types of adverse health effects differ by levels and types of air pollutants. This information is needed so that source specific interventions can be developed to mitigate and prevent the adverse health effects of air pollution on the ocular surface, particularly in the indoor air environment as its quality is not regulated by the EPA.

## 2. Methods

**Figure 2:**
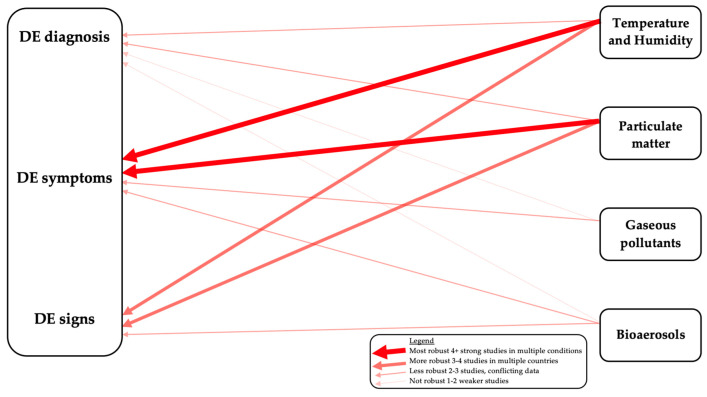
Associations between various environmental exposures and dry eye (DE) diagnosis, symptoms, and signs with the size of each arrow indicating the strength of the existing data.

A PubMed search was conducted using the key terms “dry eye” AND either “temperature”, “humidity”, “controlled chamber”, “pollution”, “mold”, “ozone”, “nitrogen dioxide”, “sick building syndrome” and “eye” AND “volatile organic compounds”. All published primary scientific articles were considered, and review articles were excluded. All searches were limited to the English language. Eligible articles were reviewed and summarized. We organized our review by exposure type (particulate matter, gaseous pollutants, and weather) although we are cognizant of overlaps in these categories. We further organized each section by exposure location (outdoor vs. indoor) and DE sub-types (diagnosis, DE symptoms, and DE signs). We chose these groupings as clinically, DE symptoms and signs are often discordant [14,15], suggesting that different contributors may underlie different DE manifestations. In addition, mitigation approaches differ between indoor and outdoor exposures. We summarize our interpretations on the strength of relationships between different exposures and DE parameters in Figure 2.

## 3. Dry Eye as a Disease Model

Dry eye (DE) is a prevalent, chronic disease that is often encountered by both eye care and primary care providers [16]. DE is defined as a multifactorial disease of the ocular surface classified by a loss of homeostasis of the tear film that may be accompanied by ocular symptoms. Tear film instability and hyperosmolarity, ocular surface inflammation and damage, and neurosensory abnormalities are hypothesized to play etiological roles [17]. DE has been referred to as a “vicious cycle” as an initial insult (e.g., allergy, environmental factors, surgery, Meibomian and lacrimal gland dysfunction) propagates inflammation, an unstable tear film, and apoptosis that perpetuates symptoms and signs of DE even if the initial insult is gone [18]. Symptoms of DE include pain characterized as dryness, discomfort, irritation, burning, itchiness, stinging, foreign body sensation, or light sensitivity and visual complaints such as unstable or poor quality vision [19]. In humans, DE is primarily first identified through symptoms. However, objective signs of DE include decreased tear film stability, decreased aqueous tear production, elevated or unstable tear osmolarity, and inflammation on the ocular surface. DE symptoms are usually assessed through standardized questionnaires such as the Ocular Surface Disease index (OSDI) [20] and DE signs through objective tests such as measurement of TBUT to evaluate tear stability, lissamine green or fluorescein staining to evaluate epithelial disruption, and Schirmer test to measure tear production [21]. Oftentimes, DE symptoms and signs do not correlate with each other, and this complicates diagnosis and management [14,15].

## 4. Effect of Particulate Matter (PM) on the Ocular Surface

### 4.1. Outdoor PM

#### 4.1.1. The Effect of Outdoor PM on a Dry Eye Diagnosis

Epidemiological studies have examined the impact of outdoor air pollution on a DE diagnosis. In one study using data from the National Veterans Administration (VA) database (*n* = 3.41 million patients), DE cases were defined as individuals with an International Classification of Disease (ICD)-9 code for DE (375.15) and medication use (e.g., artificial tears) or procedure (e.g., punctal plugs) for DE [22]. Aerosol optical depth (AOD) was used as an indicator for concentration of aerosols and served as a surrogate measure of air pollution. Higher levels of AOD (measured at each treatment facility) elevated the risk for a DE diagnosis (1.126, 95% CI = 1.125–1.127, *p* < 0.001). Using the same sampling strategy, the diagnosis of DE was found to have a seasonal pattern with peaks in winter and spring, with the highest prevalence in April (20.9 ± 0.14%), coinciding with elevated pollen indices from www.pollen.com [23]. In a Taiwanese study of 25,818 subjects with DE identified by ICD-9-CM codes from a 1,000,000 person database of insured patients, the first occurrence of DE was also the highest in spring and early summer (26.2% and 25.4%), again aligning with the allergy season [24].

Air pollution has also been linked to a DE diagnosis in Asia. In a Chinese retrospective case-crossover study, 5062 individuals were identified as having an outpatient visit for DE by ICD-10 codes [25]. Daily concentrations of PM_2.5_ and PM_10_ were collected by eight urban background air quality monitors covering the city districts of Hangzhou. Using a single pollutant model with subjects serving as their own controls (on the same weekday within the same month), same day exposure of PM_2.5_ (1.02, 95% CI = 1.01–1.03, *p* < 0.01) and PM_10_ (1.01, 95% CI = 1.003–1.02, *p* < 0.01) increased the risk of an outpatient DE visit. Additionally, PM_2.5_ and PM_10_ had a lasting effect on DE diagnosis up to 2 and 3 days, respectively, after exposure using a lag model, with no significant change after 3–7 days. In another Chinese cross-sectional study, 23,922 subjects were recruited from ophthalmic clinics in 32 cities [26]. DE was defined by the presence of ≥1 symptoms such as dryness, foreign body sensation, burning, eye fatigue, discomfort, or vision fluctuation, and one abnormal sign such as a TBUT ≤5 s, presence of fluorescein staining, or Schirmer test ≤ 5 mm/5 min. Pollution levels were considered “extreme” if the daily average PM_2.5_ level for a specific city was greater than 81.21 μg/m^3^. Extreme pollution levels for >143 days in a year increased the risk of a DE diagnosis by 2.01 (95% CI = 1.79–2.25; *p* < 0.0001).

In summary, several US and Asian studies have found a link between a DE diagnosis and air pollution [22,25]. However, one big limitation in using ICD codes to diagnose DE is the inability to examine which facet of DE is related to air pollution.

#### 4.1.2. The Effect of Outdoor PM on Dry Eye Symptoms

DE symptoms have been linked to air pollution. In an Indian study, 500 healthy health care workers answered questions regarding the presence of eye redness, irritation, tearing, blurred vision, photophobia, eye strain, burning, and dryness [27]. Of note, levels of suspended PM in New Delhi are five times (~210–360 µg/m^3^) higher than the annual average level set by the WHO (60 µg/m^3^). Individuals were divided based on mode of traveling to the hospital: (a) 250 individuals who commuted to the hospital via open vehicle (e.g., scooter, bicycle, or motorcycle) and (b) 250 individuals who lived on the medical campus and walked to work. Individuals who commuted had higher frequencies of redness (42% vs. 20%), irritation (50% vs. 26%), tearing (38% vs. 18%), burning (28% vs. 0%), and dryness (8% vs. 0%) compared to those who walked to work, *p* < 0.05 for all. Interestingly, visual quality was similar in both groups. The underlying assumption of this study is that individuals who commute are exposed to higher levels of air pollution than those that do not commute. However, a limitation of the study is that commuters are likely exposed to a variety of environmental insults (PM, gaseous pollutants, weather conditions), and thus, the effect of PM on DE cannot be evaluated in isolation. In contrast to the New Delhi findings, in a prospective study from Thailand, 3025 participants answered 19-yes/no questions regarding various eye symptoms daily for 4 months [28]. All subjects lived within a 10-km radius of an air quality monitoring station where ambient PM_10_ levels were continuously measured. In this study, PM_10_ was positively associated with blurred vision (OR = 1.009; 95% CI = 1.004–1.014) but not with irritation or redness.

Furthermore, not all studies have reported a link between air pollution and DE symptoms. In a cross-sectional South Korean study, 16,824 subjects from The Korea National Health and Nutrition Examination Survey (KNHANES) answered the questions: “Do your eyes frequently feel dry or irritated?” and “Have you ever been diagnosed by an ophthalmologist as having dry eye syndrome?” [29]. Of the participants, 21.7% answered yes to having symptoms and 11.8% to having a diagnosis. Air pollution data were pulled from 283 atmospheric monitoring stations located throughout South Korea over a 3-year period using each participant’s residential division as a proxy for their location. Levels of PM_10_ were not associated with DE symptoms (R = −0.032, 95% CI, −0.313 to 0.254, *p* = 0.83) or a diagnosis (R = 0.1080, 95% CI, −0.3805 to 0.1818, *p* = 0.47). Similarly, in a Brazilian study, 71 healthy male taxi drivers or traffic controllers carried a passive sampling device to assess PM_2.5_ concentration [30]. Individuals were then seen in the eye clinic on 4 occasions where they underwent an eye examination, filled out the OSDI questionnaire, and levels of PM_2.5_ were measured. At visit 1, mean OSDI score was 9.18 ± 6.81, which is relatively low as the OSDI ranges from 0–100. There was no significant change observed in OSDI scores throughout the study although the subjects were exposed to high levels of PM_2.5_ (40 mg/m^3^).

Other studies have focused on particular PM types. In a Swedish study, 89 children aged 7–18 with known pollen allergy (positive skin prick test or specific IgE) graded their eye symptoms from 0 “no symptoms” to 3 “severe symptoms” each day for 42 days [31]. Pollen levels were monitored through a centrally located rooftop volumetric spore trap both in and out of pollen season. DE symptoms scores increased linearly with pollen grain exposure until about 150 grains/cm^3^ where the curve seems to flatten out. Overall, ocular symptoms were significantly dependent on pollen count throughout the study period.

In summary, some [27,28], but not all [29,30], studies have found a associations between DE symptoms and outdoor PM. Furthermore, there are inconsistencies between studies regarding the types of symptoms that relate to air pollution (pain vs. visual disturbance).

#### 4.1.3. The Effect of Outdoor PM on Dry Eye Signs

Several studies have reported on relationship between outdoor PM and DE signs, but again, with variability in findings across studies. In the New Delhi study [27], individuals who commuted had more rapid TBUT and less tear production than individuals who walked to work (TBUT: 12.97 ± 6.12 vs. 19.23 ± 5.70 s, *p* < 0.001; Schirmer’s test 13.42 ± 6.67 mm vs. 15.95 ± 6.14 mm, *p* < 0.001); however, values in both groups were in the normal range, and thus, the clinical significance of these differences is not clear.

Similar data have been found in South American studies. In an Argentinian study of individuals living in an industrial (*n* = 34) vs. urban (*n* = 44) zone, annual PM_10_ levels obtained from sampling sites significantly varied between groups (52.2 ± 16.7 μg/m^3^ vs. 31.9 ± 13.9 μg/m^3^, *p* < 0.05) [32]. DE signs were defined as TBUT < 10 s, corneal staining >grade 3, lid parallel conjunctival folds >grade 3, or Schirmer ≤ 10 mm/5 min. A higher percentage of individuals had ≥3 signs in the industrial vs. urban zone (61.7% *n* = 21 vs. 25%, *n* = 11, *p* < 0.001). In the Brazilian study of 71 healthy male taxi drivers or traffic controllers [30], generalized estimating equation analysis revealed that each 10 μg/m^3^ increase in PM_2.5_ resulted in a 10.9 mOsm/kg (*p* < 0.05) decrease in tear osmolarity with a dose–response pattern. However, the clinical significance of this finding is unclear as DE has been associated with increased or unstable (but not lower) osmolarity [21]. In the Brazilian study, other DE signs, such as staining and Schirmer, were not related to PM concentration.

Overall, the association between outdoor PM and DE signs is less strong than for symptoms (Figure 2), with fewer studies available, inconsistent findings across studies, and fewer number of participants in individual studies. Furthermore, the clinical significance of the findings (e.g., lower osmolarity) is not clear [27,30]. Overall, TBUT has been more consistently linked to PM exposure, as compared to corneal staining or tear production.

### 4.2. Indoor PM

#### 4.2.1. The Effect of Indoor PM on Dry Eye Symptoms

Short term exposure to an adverse indoor environment can negatively impact DE symptoms. In a prospective study of 88 subjects exiting an older building with a higher concentration of airborne PM (24,436 particles ≥ 0.5 µm/feet^3^) and a higher number of microbial colonies (1066 colonies/m^3^) and 102 subjects exiting a newer building with a lower concentration of airborne PM (12,313 particles ≥ 0.5 µm/feet^3^) and lower number of microbial colonies (400 colonies/m^3^), DE symptoms were assessed by two yes/no questions, “Are you currently experiencing any dry eye symptoms, such as dryness, irritation, or burning sensation?” and “Have your dry eye symptoms changed since the morning?” [33]. After adjusting for confounding variables, subjects leaving the older building were more likely to report DE symptoms (OR 3.89; 95% CI = 1.21–12.5; *p* < 0.05) and worsening DE symptoms (OR 13.30; 95% CI = 2.80–63.31.60; *p* < 0.01) compared to subjects leaving the newer building.

Similar findings have been seen with regards to long term exposures. In a US study of 97 individuals, subjects filled out the OSDI questionnaire and underwent home environment evaluation to assess PM level with a handheld particle counter [34]. In multivariate analyses when adjusting for demographics, comorbidities, medications and other interaction variables, a 1 unit increase in PM_2.5_ was associated with a 1.59 increased in OSDI score (95% CI, 0.58–2.59; *p* = 0.002). Similar findings were observed in Brazil. In a cross-sectional study of 936 workers in an older, non-sealed office building with natural ventilation and 1736 workers in a newer, sealed office building with HVAC systems, the prevalence eye symptoms were studied in relation to concentration of PM and other building conditions [35]. In each building, indoor air quality (IAQ) was measured on 6 or more floors at 2 different sampling points for 8 h. Subjects completed an 11-question symptom survey that included a section on dry, itchy or watery eyes. Subjects ranked the frequency of their symptoms from 1 being “every day” to 5 being “less than every 2–3 weeks” and answered if their symptoms were better on days away from the office. PM levels were high (80–800 μg/m^3^) or very-high (>800 μg/m^3^) on all 6 floors of the older building while 3 of 7 floors in the newer building had low PM (<80 μg/m^3^). Eye dryness (33.3% vs. 27.1%, *p* = 0.01) was more frequent in the older building compared to the newer building. In addition, symptoms of itching were more likely to improve upon leaving the older vs. newer building (64.0% vs. 72.9%, *p* < 0.05).

Similarly, organic PM like bioaerosols have been associated with DE symptoms in schools and homes. In a Malaysian study of 462 middle school students, vacuumed dust levels from 32 classrooms were analyzed and studied with responses to a self-administered questionnaire [36]. Subjects rated the frequency of eye irritation in the last 3 months as occurring every day, 1–4 times a week, 1–3 times a month, or never. The level of vacuumed fine dust on the filters was associated with weekly ocular irritation (OR = 4.19; 95% CI = 2.31–7.62; *p* < 0.001). Concerning home exposures, 3485 randomly selected healthy adults in China answered questions regarding the presence of mold/damp stains, moldy odor, and water damage in the home and rated eye itchiness and burning as weekly, sometimes, or never [37]. Individuals who lived in homes with multiple signs of dampness and mold were at an increased risk for ocular itching, irritation, or burning (OR 3.20; 95% CI 1.67–6.15; *p* < 0.01).

To conclude, the link between indoor PM exposure and painful DE symptoms is strong (Figure 2) with multiple studies across different countries and building situations reporting a link between office and home PM exposure and dryness and irritation.

#### 4.2.2. The Effect of Indoor PM on Dry Eye Signs

Less information is available on the link between an adverse indoor environment and DE signs. In the study of 97 South Florida veterans [34], a one unit increase in PM_2.5_ was also associated with a 0.39 reduction in Schirmer score (95% CI, −0.75 to −0.03; *p* = 0.04), a 0.07 increase in meibomian gland dropout (95% CI, 0.01–0.13; *p* = 0.02), and a 0.06 increase in inflammation measured via Inflammadry strips (95% CI, 0.02–0.11; *p* = 0.009). In a follow up study by the same group, indoor air was sampled in the home of 157 veterans with a biostage impactor and incubated on nutrient and soy media [38]. Using a multivariate linear regression, elevated microbial counts were associated with meibomian gland drop out when adjusting for age, gender, log (total suspended particles), smoke exposure, allergy status, and number of observations ((OR 67.42, 95% CI = 1.18 to 3864.59, *p* < 0.05). Corneal epithelial disruption was significantly associated with higher numbers of colonies in the model adjusting for age and gender only (OR 28.07, 95% CI = 1.78 to 443.79, *p* < 0.05).

Similar findings were seen outside the U.S. In a study of children in Malaysia, TBUT was evaluated in relation to fungal DNA in dust collected by vacuuming 32 classrooms in 8 different middle schools [39]. Fungal DNA was associated with a 5.1 s decrease in TBUT (95% CI = −9.1 to −1.2, *p* < 0.05) per 10^5^ target copies per gram dust, linking exposure to fungus with tear instability.

Environmental chamber studies support these findings. An environmental chamber is an enclosure that can be used to test the effects of specified environmental conditions (e.g., temperature, humidity, PM exposure) on biological items (in our case, facets of DE). In a chamber study in Denmark, 10 individuals were exposed to clean (~41 μg/m^3^ dust) and polluted air (~394 μg/m^3^ dust) in a randomized order for 3 h while in a controlled chamber. TBUT was significantly impacted by dust exposure with 7 of 10 subjects having a decrease in TBUT after exposure to dust, *p* < 0.05 [40]. To conclude, while fewer studies are available, they are consistent in highlighting a link between tear instability and dust. Interesting, TBUT is also the DE sign most affected by outdoor PM, as summarized above.

### 4.3. Potential Pathophysiology

There is biologic plausibility linking air pollution to DE. First, as summarized above, some studies have found an inverse correlation between air pollution and TBUT [27,41]. In a similar manner, mouse studies have supported this clinical observation [42]. In a study of male mice, mice were exposed to either 5.0 mg/mL PM_2.5_ or saline. The PM_2.5_ group showed significantly decreased TBUT at day 14 compared to the saline group (~2.5 ± 0.75 s vs. 6.5 ± 1.6 s, *p* < 0.05). In the same study, the PM_2.5_ group were also found to have higher levels of inflammatory markers compared to the saline group (tumor necrosis factor-α (TNF-α) ~0.38 ± 0.17 vs. 0.32 ± 0.10, *p* < 0.05; NF-κB ~2.75 ± 0.6 vs. ~0.33 ± 0.1, *p* < 0.05). In fact, inflammation has been found to be a key component of DE in humans [43] and animal models [44].

Similar to the human studies [36,37,39,40], organic bioaerosols have also been reported to negatively impact ocular surface health. In an in-vitro study, human corneal epithelial (HCE) cells were exposed to various concentrations of mycotoxins (AFB_1_ and gliotoxin) vs. a solvent control, and in vitro assays were done to asses cellular viability and inflammatory responses [45]. Exposure to AFB_1_ (16 µg/mL) increased expression of several inflammatory markers (380-fold increase in IL-8, 160-fold increase in C-X-C motif chemokine ligand 1, 21-fold increase in TNF-α) compared to the control. Exposure to gliotoxin (125 ng/mL) insignificantly increased interleukin-8 (IL-8) and TNF-α expression but significantly increased toll like receptor 4 (TLR-4) expression 1.9-fold (*p* = 0.0041) and poly ADP-ribose polymerase (PARP) expression 1.4-fold (*p* < 0.0001). These data support the clinical findings that mycotoxins can trigger inflammatory responses.

While inorganic and organic PM influence inflammation on the ocular surface, inorganic PM like dust is also associated with oxidative stress. In a study evaluating the effect of house dust on HCE, dust was collected from 16 homes in Nanjing, China, from the air conditioning units [46]. Primary HCE cells were exposed to either a fresh medium containing between 5 to 320 μg/100 μL of dust or a control medium. Oxidative stress induced DNA damage was assessed by measuring accumulation of 8-OHdG. At the lower level of 5 μg/100 μL, 8-OHdG levels were similar to controls (214 ± 6.50 pg/mL). However, 8-OHdG levels greatly increased to 400 ± 38.8 pg/mL after exposure to 80 μg/100 μL of dust. In fact, a concentration-dependent relationship was revealed between house dust level and ROS production.

Together, these in-vitro and animal studies suggest that inflammatory responses, which play a large role in DE, are triggered by inorganic and organic PM. Additionally, oxidative stress is triggered by non-organic PM.

## 5. Effect of Gaseous Pollutants on the Ocular Surface

### 5.1. Outdoor Gaseous Pollutants

#### 5.1.1. The Effect of Outdoor Gaseous Pollutants on a DE Diagnosis

Gaseous pollutants include reactive gases such as NO_2_, SO_2_, and O_3_ and VOCs such as formaldehyde, toluene, acetone, ethanol, and more that are present in both in the outdoor and indoor environment. These pollutants have various sources including car and fuel emissions, wood preservatives, aerosol sprays, disinfectants, combustion, cigarette smoke, and many other sources that may impact human health. Outdoor exposure to reactive gases has been linked to a DE diagnosis but with discrepancies across studies in relation to the specific gases involved, making the data less robust (Figure 2). In a case-crossover study in Taiwan [24], multivariable analyses found significant associations between NO_2_ (OR 1.07; 95% CI = 1.04–1.10; *p* < 0.001) and CO (OR 1.12; 95% CI = 1.03–1.21; *p* < 0.01) and a DE diagnosis. Specifically, every 10-ppb increase in NO_2_ was associated with a 6.8–7.5% increase in a DE diagnosis and every 1-ppm increase in CO was associated with a 10.5–11.6% increase in a DE diagnosis. In another study in Hangzhou, China [25], NO_2_ (1.03; 95% CI = 1.01–1.05; *p* < 0.05), SO_2_ (1.07; 95% CI = 1.02–1.11; *p* < 0.01) and CO (1.0; 95% CI = 1.0–1.0; *p* < 0.01) were associated with DE outpatient visits in a multi-pollutants model. O_3_ did not significantly associate with DE. Other studies, however, have reported associations between O_3_ and a DE diagnosis. In a South Korean population-based cross-sectional study [29], using multivariable models, a 0.003-ppm increase in O_3_ level was associated with both a diagnosis and symptoms of DE (OR 1.31; 95% CI = 1.12–1.53; *p* = 0.002). A 0.003-ppm increase in the NO_2_ level was associated with a diagnosis of DE but not symptoms (OR 1.12; 95% CI = 1.02–1.23; *p* = 0 .02), and SO_2_ was not associated with either DE diagnosis or symptoms (OR 1.10; 95% CI = 0.84–1.43; *p* = 0.50). There is a paucity of literature relating VOCs to DE diagnosis.

#### 5.1.2. The Effect of Outdoor Gaseous Pollutants on DE Symptoms

Outdoor exposure to gaseous pollutants has been linked to DE symptoms, again with some inconsistencies in the data. Associations of gaseous pollutants to symptoms are stronger than to diagnosis and signs but still weaker than to other pollutants (Figure 2). In a study from Chiang Mai, Thailand [28], NO_2_ was positively associated with eye irritation (OR 1.05, 95% CI = 1.03–1.07 per 1 ppb), and SO_2_ was positively associated with eye irritation, red eyes, and blurred vision (OR 1.26, 95% CI = 1.09–1.47, 2.95, 95% CI = 1.46–5.94, and 1.21, 95% CI = 1.0–1.46 per 1 ppb, respectively). Conversely, O_3_ was negatively associated with red eyes and blurred vision (OR 0.89, 95% CI = 0.85–0.94 and 0.962, 95% CI = 0.95–0.98 per 1 ppb, respectively). However, O_3_ was linked to DE symptoms in other studies. In a prospective observational study of 33 individuals in South Korea with evaporative DE (TBUT ≤ 5 s) who were using topical drugs (0.05% cyclosporine or 3% diquafosol), DE measures were investigated twice at 2-month intervals, and outdoor O_3_ concentrations on the clinic visit days were obtained from public atmospheric monitoring stations and calculated based on each subject’s address [47]. In multivariable models, Ocular Surface Disease Index (OSDI) scores increased by 3.43 points per each 0.01 ppm increase in O_3_ concentration (b = 3.43, *p* = 0.002). A limitation of this study is that it is not clear which symptoms within the OSDI (pain vs. visual disturbance) drove the increased OSDI scores.

Furthermore, not all studies have linked gaseous exposure with DE symptoms. In a Brazilian study of 71 taxi drivers and air traffic controllers [30], NO_2_ was continuously measured through passive sampling tubes for 24 h and subjects filled out an OSDI questionnaire the same morning as when the air pollutant samplers were removed. While subjects were exposed to a mean NO_2_ level of 189 ± 47 μg/m^3^, OSDI scores were relatively low (7.4 ± 8) and were not correlated with NO_2_ levels.

Outdoor VOCs have also been studied in relation to DE symptoms. For example, a study in Neyagawa, Osaka, Japan, compared 6217 individuals living within 1200 m from a plastic recycling factory to 77 individuals living 2800 m away from the factory [48]. Concentrations of VOCs were measured at two locations, 100 and 500 m from the factory, for 3 h/day on 3 days of the same week (June 2006), using an automatic thermal desorption tube. Unsurprisingly, the location closer to the factory had higher concentrations of VOCs than the location further away. Self-administered questionnaires assessed whether subjects had eye itch, redness, irritation, and discharge more than once per month. Several symptoms were more frequent in residents within 500 m of the factory compared with 2800 m from the factory including itch (OR = 3.0, 95% CI: 1.5–6.0), redness (OR = 2.7, 95% CI: 1.1–6.3), irritation (OR = 5.2, 95% CI: 1.5–17.8), and discharge (OR = 6.0, 95% CI: 2.3–15.9). In another study performed on 111 individuals living near a petrochemical industrial estate in Thailand, VOC data were obtained from an ambient air monitoring station of the Pollution Control Department of the Ministry of Natural Resources and Environment, located at the primary care unit in one of the communities in the study area [49]. Individuals were interviewed daily for 63 days about the presence of twelve symptoms, categorized into respiratory symptoms and other health symptoms (headache, shortness of breath, fever, eye irritation, dizziness, and weakness). The interviewers asked questions about symptoms on the previous day. A 1 ppm increase in total VOCs was associated with an increased odds of eye irritation (OR 4.30, 95% CI 14–16.26, *p* < 0.05). Taken together, these data highlight the inconsistency in the relationships between reactive gases and DE. For example, while associated with a DE diagnosis, NO_2_ and SO_2_ were not found to associate with DE symptoms. Additionally O_3_ was associated with DE symptoms in two Asian studies [29,47] but was protective for DE symptoms in another Asian study [28]. Furthermore, most studies used data from monitoring stations and thus do not necessarily reflect individual level exposures. These studies also suggest a relationship between outdoor VOC exposure and DE symptoms, although unaccounted confounders (e.g., PM) may also contribute to the noted relationships.

#### 5.1.3. The Effect of Outdoor Gaseous Pollutants on DE Signs

Only a few studies have examined relationships between outdoor gaseous pollutants and DE signs. In one study, 29 hospital employees from two cities in Brazil with varying levels of air pollution (São Paulo and Divinolândia) underwent conjunctival sampling via impression cytology [50]. Mean individual levels of NO_2_ were measured using a passive sampler for 7 days. Exposure to NO_2_ was stratified into four quartiles (Q) based on NO_2_ levels: Q1, <17 μg/m^3^; Q2, 18–21 μg/m^3^; Q3, 22–33 μg/m^3^; and Q4, >33 μg/m^3^. Conjunctival goblet-cell counts increased proportionately to NO_2_ exposure: Q1, 186 cells; Q2, 253 cells; Q3, 312 cells; and Q4, 402 cells. Furthermore, NO_2_ concentrations were positively correlated to goblet cell counts per 10 high power field (ρ = 0.57, *p* = 0.001). In addition to number, goblet-cell hyperplasia was also positively related to NO_2_. However, the clinical significance of this finding in relationship to DE symptoms and signs is not clear. Furthermore, as with the New Delhi study, there are likely other differences between the populations (PM, weather) that were not accounted for. In the Korean study [47], short-term changes in ground-level O_3_ were associated with decreased tear secretion (decrease of 1.43 mm per 0.01 ppm increase in O_3_ concentration, *p* = 0.015), but again, the clinical significance of this findings is unclear. Taken together, these studies highlight the paucity of literature on associations between gaseous pollutants, especially VOCs and ocular surface signs (Figure 2), but also suggest that tear production and goblet cell density may be affected by exposure to reactive gases.

### 5.2. Indoor Gaseous Pollutants

#### The Effect of Indoor Gaseous Pollutants DE Symptoms

Of all gaseous pollutants, we found a greater number of studies on indoor O_3_ exposure in relation to DE symptoms. O_3_ is often studied indoors through aircraft cabins as there are elevated levels of O_3_ at high altitude [51]. Cabin air of 83 flights was monitored between 2008–2010 as a part of a study of onboard environmental conditions. O_3_ measurements were conducted by a project engineer onboard the aircraft. Mean O_3_ concentration across all flights was 16.1 ppb. Approximately 52 passengers per flight filled out a yes/no survey for a range of symptoms on board including itching eyes, dry eyes, and watery eyes. Although 16.1 ppb is lower than the EPA guideline, O_3_ concentration was significantly associated with itchy eyes (OR 1.16; 95% CI = 1.04–1.29; *p* = 0.008), but not dry or watery eyes. Simulated cabin studies similarly revealed an association between DE symptoms and elevated ozone levels [52]. In a study of 29 females in Denmark, subjects were split into two groups and exposed to two levels of O_3_ (<2 and 60–80 ppb) at two different air flow rates (2.4 vs. 4.7 L/s). Subjects graded eye irritation from 0 being no irritation to 50 being overpowering irritation quickly after entering and after 3.25 h in the chamber. Eye irritation was higher in the ozone group (60–80 ppb) at both flow rates but only significantly at the high flow rate (7.2 vs. 2.5, *p* < 0.015). These findings are in agreement with the Korean study [47] that found a relationship between outdoor O_3_ and symptoms, but in contrast to the Thailand study [28] that found the outdoor O_3_ was protective for symptoms.

Indoor NO_2_ level has also been associated with DE symptoms. In a study in Malaysia of 462 s grade students, weekly symptoms of eye irritation in the last month were compared to NO_2_ level inside and outside the classroom for 7 days using a diffusion sampler [53]. The presence of eye irritation (yes/no) was positively related to indoor NO_2_ concentration (OR 2.34, 95% CI 1.40–3.90; *p* = 0.001). These findings are in agreement with the Thailand study [28] that found a relationship between outdoor NO_2_ and symptoms, but in contrast to the Brazilian study [30] that found no correlation between NO_2_ and symptoms.

Indoor exposure to VOCs has also been associated with DE symptoms. For example, a study conducted in Heidelberg, Germany, on 21 subjects investigated the occurrence of eye symptoms after exposure to 10 different formaldehyde concentrations. Subjects were exposed to each of the 10 concentrations for 4 h on 10 consecutive days, in random order. Eye irritation (graded on a 0–5 scale) at exposure levels as low as 0.3 ppm was significantly higher than at 0 ppm after 195 min of exposure (0 ppm exposure ~0.3 symptoms score vs. 0.3 ppm exposure ~0.6 symptoms score after 195 min; *p* < 0.05) [54]. In support of the experimental study, a South Korean study assessed 34 subjects when they moved from a rehabilitation hospital with low indoor VOCs to a building with higher VOC levels (benzene, toluene, ethylbenzene m, *p* xylene, styrene, o-xylene, formaldehyde, and acetaldehyde, all measured in both buildings) [55]. Severities of eye dryness and eye irritation were found to increase via 0–10 visual analogue scale score with the move to the new building, potentially linking VOC to DE symptoms.

Exposure to VOCs can also be assessed through urine levels. In a study of 389 employees in 8 high-rise buildings in Taipei, eye dryness (yes/no) was subjectively assessed while 8-hydroxydeoxyguanosine (8-OHdG) was quantified in urine (a marker of oxidative stress related to VOC levels) [56]. Individuals who reported eye dryness had higher levels of 8-OHdG in urine (6.16 ± 3.81 vs. 5.45 ± 3.10 μg/g; *p* = 0.047), supporting a relationship between symptoms and VOC exposure.

VOCs have been linked to DE symptoms in children as well. In a Malaysian study of middle school children [53], VOC level inside and outside the classroom were sampled for 7 days using a diffusion sampler and the frequency of eye irritation was assessed. The presence of eye irritation (yes/no) was positively related to indoor formaldehyde concentration (OR 3.55, 95% CI 1.51–8.38; *p* = 0.004).

VOCs in the home have also been associated with DE symptoms. A Japanese study randomly selected 33 homes where at least one inhabitant complained of one or more eye symptoms (eye irritation, dry eyes, photophobia, lacrimation, and eye redness) and 63 homes where none of the inhabitants complained of symptoms [57]. Concentration of 19 different VOCs and total VOCs was measured by passive samplers located in the living room over a 1-week period, and self-administered questionnaires were assessed for the presence of eye symptoms. Indoor concentrations of ethylbenzene (OR 2.1, 95% CI 1.07–4.10; *p* <0.05), xylene (OR 2.29, 95% CI 1.11–4.69; *p* <0.05), and total VOCs (OR 9.79, 95% CI 1.67–57.5; *p* <0.05) were found significantly related to eye symptoms.

To summarize, while studies have found links between indoor O_3_ and NO_2_ with DE symptoms, there is a lack of data on the effects of other gases like indoor SO_2_ and CO on DE. Furthermore, studies are needed to evaluate relationships between indoor reactive gas exposure and DE signs. The data linking outdoor and indoor VOCs in a variety of locations (workplace, school, home) to DE symptoms are stronger than for other gases, but there is a paucity of literature relating VOCs to DE signs.

### 5.3. Potential Pathophysiology

The mechanisms by which gaseous pollutants may affect the ocular surface health can be inferred by data generated outside the eye where it has been demonstrated exposure to reactive gases can lead to cell death, oxidative stress, and inflammation [58,59]. For example, human alveolar macrophages were exposed to 0.4 ppm concentration of O_3_ for 1 h as compared to purified air with 5% CO_2_ at a rate of 2 L/min [60]. Exposure to 0.4 ppm O_3_ increased the secretion of inflammatory cytokines such as TNF-α, IL-lβ, IL-6, and IL-8 compared to control (% increase vs. control: TNF-α 443% ± 208; IL-lβ 484% ± 171; IL-6 383% ± 147; IL-8 242% ± 63). In another study, 11 healthy male volunteers were exposed on two separate occasions (at least 6 weeks apart) to filtered air and 0.4 ppm of O_3_, with bronchoalveolar lavage (BAL) conducted the following morning [61]. An increase in percentage of polymorphonuclear leukocytes (PMN) and decrease in macrophages in BAL fluid were found after O_3_-exposure (mean ratio 8.2 ± 2.2; *p* = 0.009 and mean ratio 0.97 ± 0.02; *p* < 0.001, respectively). Moreover, markers of inflammation in BAL fluid were higher after O_3_ exposure versus air exposure including fibronectin (mean ratio 6.44 ± 1.54, *p* = 0.006), complement C3a (mean ratio 1.72 ± 0.14, *p* = 0.001), and prostaglandin E_2_ (PGE2) (mean ratio 1.97 ± 0.21, *p* = 0.002).

When considering the effects of O_3_ on the ocular surface, one study exposed 30 male mice to filtered room air (controls), 0.5 ppm, or 2.0 ppm of O_3_ for 3 h every day for 2 weeks in a whole-body exposure chamber [62]. After 2 weeks, mice exposed to 2 ppm O_3_ had higher levels of inflammatory cytokines in tear fluid compared to 0.5 ppm exposure and controls, including IL-1β (2 ppm ~ 875 pg/mL vs. 0.5 ppm ~ 300 pg/mL; *p* < 0.001), IL-6 (2 ppm ~ 800 pg/mL vs. 0.5 ppm ~ 225 pg/mL; *p* < 0.001), IL-17 (2 ppm ~ 775 pg/mL vs. 0.5 ppm ~ 250 pg/mL; *p* < 0.001), and IFN-γ levels (2 ppm ~ 875 pg/mL vs. 0.5 ppm ~ 250 pg/mL; *p* < 0.001).

Moreover, when studying NO_2_ effects, various studies support a positive correlation between exposure and eosinophilic airway inflammation in asthmatics. For example, a study investigated repeated brief exposures to concentrations of 200 and 600 ppb NO_2_, to simulate indoor NO_2_ fluctuations and its correlation with airway inflammation through induced sputum samples in 19 asthmatic adults. They found an association between the changes in percentage of eosinophils and NO_2_ exposure levels relative to baseline measurements was significant (0 ppb NO_2_: −12 (−34, 16), 200 ppb NO_2_: −5 (−28, 26), 600 ppb NO_2_: 57 (18, 109), *p* = 0.04) [63].

Additionally, VOCs have been linked to harmful immunologic, respiratory, carcinogenic, reproductive, neurologic, and cardiovascular health effects. Potential mechanisms are oxidative stress, inflammation, and cell death [64]. For example, a study investigated the effects of cigarette smoke (a source of VOCs) on mouse and human lung epithelial cells. Exposure to up to 3 puffs of cigarette smoke resulted in a 10% mortality in mice cells, and exposure to 7 or more puffs resulted in an 80% mortality in mice cells. Interestingly, when VOCs, were removed from the cigarette smoke, no cell death was seen, even with the same CO and NO concentrations, supporting the idea that VOCs are responsible for direct cytotoxic in cells, and further supporting the noted clinical relationships [65]. Similar to PM, associations between gas exposure and inflammation may be an important link connecting O_3_ and other gaseous pollutants to DE.

## 6. Effect of Weather on the Ocular Surface

### 6.1. Outdoor Weather Conditions

#### 6.1.1. The Effect of Outdoor Weather Conditions on a Dry Eye Diagnosis

Environmental factors such as humidity, wind, and altitude affect the risk of DE. Ultimately, there is a general acknowledgement that extremes of temperature and humidity are not ideal for the eye, and it appears that moderate temperature and humidity with minimal wind and UV light are optimal. In the retrospective analysis using data from a National Veterans Administration (VA) database [22], spatial information was recorded based on treatment facility, and using a statistical trend analysis, the risk of DE was higher in zip codes with an atmospheric pressure 1 standard deviation (SD) above the mean (IRR, 1.131; 95% CI, 1.129–1.133; *p* < 0.001). Conversely, high humidity and wind speed lowered the risk of DE (IRR, 0.927 [95% CI, 0.926–0.927] and IRR, 0.938 [95% CI, 0.937–0.939], respectively; *p* < 0.001).

Other studies have supported the protective association between higher outdoor humidity and DE symptoms. In a cross-sectional study of a South Korean population [29], multiple logistic regression analyses revealed a negative association between DE diagnosis and relative humidity levels (R = −0.425 (95% CI, −0.633 to −0.161), *p* = 0.003). In another Korean study of 16,431 subjects who participated in the KNHANES, 1616 individuals had a previous diagnosis of DE, and 2666 responded positive to having DE symptoms (1314 had both). Environmental information was extracted from the National Climate Data Service System where temperature and humidity were assessed every 8 h each day. Wind speed was measured by using the total value of artistic air divided by 86,400 s, and sunshine was defined by hours of sun rays without blockage. Using multiple logistic regression analyses, a DE diagnosis was negatively associated with humidity levels (OR 0.970; 95% CI 0.955–0.986) and positively with temperature (OR 1.076; 95% CI 1.009–1.148) and sunshine duration (OR 1.015; 95% CI 1.006–1.023) [66].

While humidity appears protective for a DE diagnosis, other weather conditions such as sunlight and wind appear harmful. In a study in India, 500 eye hospital patients answered 13 yes/no questions regarding eye symptoms, and a total symptom score was calculated [67]. Self-reported environmental exposures (sunlight, high temperatures, excessive winds) were also assessed. An ocular surface examination was then performed (TBUT, staining, Schirmer’s test). A subject was defined as having DE if they had 3 or more positive tests. A DE diagnosis was associated with sunlight (OR: 1.91, *p* = 0.014) and excessive winds (OR: 2.15, *p* = 0.004).

High altitude is another risk factor for DE diagnosis. In one study, 100 subjects living in the Leh district of Ladakh (3300 m above sea level) and 100 age and sex matched subjects living in New Delhi (218 m above sea level) were recruited and screened for DE symptoms using McMonnies’ Questionnaire (MMI) and OSDI, and a Schirmer test was performed [68]. DE was defined as an MMI > 14, OSDI > 23, and Schirmer < 10 mm. The prevalence of DE was higher in individuals living in high altitude vs. low altitude (20% vs. 9%, *p* = <0.005). Together, several studies provide strong data that higher outdoor humidity protects against a DE diagnosis while high altitude, sunlight, and high temperatures are risk factors for disease. Wind associated with DE diagnosis in some [67], but not all studies [22].

#### 6.1.2. The Effect of Outdoor Weather Conditions on Dry Eye Symptoms

Weather conditions have also been associated with DE symptoms. In the South Korean study [29], multiple logistic regression analyses revealed that DE symptoms negatively correlated with relative humidity level (R = −0.411 (95% CI, −0.622 to −0.144), *p* = 0.004). In another Korean study, DE symptoms positively correlated with sunshine duration (OR 1.013; 95% CI 1.005–1.021) and negatively with humidity (OR 0.975; 95% CI 0.959–0.991) [66].

As with a diagnosis, factors such as high wind, sunlight, and heat have been linked with DE symptoms. In an observational survey-based study of 5 European countries, individuals ≥40 years using daily tear substitutes with a diagnosis of DE or Sjogren’s by a healthcare professional (*n* = 738) filled out a 26-question survey on DE symptoms and exacerbations [69]. By self-report, the environmental factors that most impacted DE symptoms were wind, sunlight, and heat, affecting 71%, 60%, and 41% of respondents, respectively. In fact, only 8% of individuals did not report that at least one facet of weather impacted DE symptoms. Overall, many studies support the association between outdoor weather conditions to both a DE diagnosis and DE symptoms (Figure 2).

#### 6.1.3. The Effect of Outdoor Weather Conditions on Dry Eye Signs

Fewer studies have examined the link between outdoor weather condition and DE signs. However, one study evaluated 200 subjects living at low and high altitudes in India. Schirmer scores were lower in individuals living at high vs. low altitudes (15.22 vs. 21.01, *p* = 0.001) [68]. However, since mean values of both groups were in the normal range, the clinical significance of this finding is uncertain. In another study of 535 U.S. dry eye patients identified by the presence of ocular dryness for greater than 6 months with moderate to severe OSDI scores (25–80), multivariate analyses and partial correlation coefficients revealed TBUT measurements were positively correlated with temperature, humidity, and dew point (ρ = 0.08, 0.18, 0.17, respectively, *p* < 0.0038) [70].

### 6.2. Indoor Temperature and Humidity

#### 6.2.1. The Effect of Indoor Humidity and Temperature on Dry Eye Symptoms

Similar to findings outdoor, low humidity has also been associated with DE symptoms indoor. In a Finnish study, 290 office workers located in two wings of the same building participated in a 6-week study where relative humidity (RH) conditions were “crossed-over” [71]. One week, individuals worked in high humidity conditions (30–40% RH) while the next week, they worked in unmanipulated “natural” conditions (20–30%). Air temperature was consistently kept between 21.3–23.9 °C. Each subject experienced three periods of exposure to the conditions. Daily self-reported eye dryness, irritation, and itching severity scores (range of 0 to 3) were all higher while working in the low vs. high RH conditions (0.39 vs. 0.35, *p* < 0.05).

High temperature indoors can also negatively affect DE symptoms. In one study, 396 U.S. office workers working on two floors of the same building had DE symptoms assessed weekly via a graded health symptom questionnaire (symptoms graded on a scale of 0–25) [72]. Environmental measurements of temperature and humidity were logged continuously with RH values between 42–58%. Continuous time-weighted data for the space each subject was working in were compared to the health symptom questionnaire. A 1 °C decrease in temperature (range 22.2–25.6 °C) was found to decrease the severity of eye dryness, itching, and irritation by 19% (95% CI ~ 8–31%).

Perception of indoor air quality has also been linked to DE symptoms. In Japan, 3335 employees in 320 offices were asked to evaluate eye symptoms and factors related to their work environment [73]. For each symptom, subjects had to respond how often that symptom occurred in the last 4 weeks and whether it persisted after leaving work. The work stressor surveys used a Likert scale (range of 1 to 4) to inquire about perceived job stress about workload and other environmental factors. Multivariable logistic regression analyses found that coldness perception (OR, 1.28; 95% CI, 1.13–1.45) and air dryness perception (OR, 1.61; 95% CI, 1.42–1.82) were associated with increased risk of eye irritation.

Controlled environment chambers studies support results of the observational studies. In one study of 10 minimally symptomatic contact lens (CL) wearers, subjects were exposed to a “adverse” environment of 22.0 ± 2.0 °C and 19.0% ± 4.0% RH for two hours [74]. Comfort was rated by subjects with 0 as extreme discomfort and 10 as extreme comfort. DE related symptomatology was evaluated using a symptom of discomfort questionnaire (SODQ) scale from 0 to 4 (0 = no symptoms, 4 = severe symptoms). Expectedly, comfort level significantly changed in CL wearers from before (9.0 ± 0.2) and after the adverse environment (8.0 ± 0.3, *p* < 0.05) in a short time period of 2 h. However, SODQ scores were not significantly impacted by the adverse environment, possibly due to the narrow scale of the SODQ compared to the more sensitive comfort scale.

Conversely, increasing local humidity has been found to improve DE symptoms. In one study, individuals with an evaporative DE (symptoms, TBUT ≤ 5 s, meibomian gland number ≤ 4 across lower eyelid) were placed in either intact swim goggles (*n* = 100) or swim goggles with the central lens removed (*n* = 25) for 20 min [75]. Individuals were asked to rate comfort 15 min after wear on a 0–4 scale. Of the subjects in intact swim goggles, 99% reported increased eye comfort compared to 24% in the control group (*p* < 0.0001). In another goggle study, 14 individuals with a aqueous tear deficiency DE (symptoms, TBUT ≤ 5 s, staining > 3, Schirmer test < 5 mm) were exposed to wind from a fan 3 m away for 10 min without spectacles, with conventional spectacles, and with spectacles that maintained a moist chamber with a humidity sensor and a humidity provider tank [76]. Subjects were examined on the same day with at least 2 h between each experimental setting. Dryness visual analog scale (VAS) scores significantly increased after wind exposure from ~20 ± 15% to ~35 ± 10% while not wearing spectacles and from ~15 ± 5% to ~30 ± 2.5% in conventional spectacles (*p* < 0.05). With moist chamber glasses, dryness scores were stable after wind exposure.

Manipulating humidity in the workplace can also improve eye comfort levels. In one study, 44 individuals in New Zealand were given desktop humidifiers (which increased RH by 5.4 ± 5.0%) and were asked to use their computers continuously for an hour [77]. Participants underwent two masked testing sessions, one with the humidifier on and one with it off. After one hour of humidifier exposure, subjects were asked if they experienced greater, equal or lesser comfort compared to baseline. Of subjects exposed to the humidifier, 36% reported an improvement in subjective comfort compared to 5% in the non-humidifier group (*p* < 0.001). Seven per cent of subjects exposed to the humidifier reported a decrease in subjective comfort compared to 48% in the non-humidifier group (*p* < 0.001).

However, not all studies have found an inverse relationship between DE symptoms and humidity. In a US study of 97 individuals with a wide range of DE metrics, a positive association was noted between humidity and DE symptoms (OSDI: r = 0.30, 95% CI, 0.07–0.49, *p* = 0.01) [34]. However, this finding was attributed to the interaction between humidity and PM and not as a direct effect of humidity on DE symptoms. That is because higher humidity can also increase the time that PM stays airborne, as well as increase PM mass and size (hygroscopic effect) [78]. This suggests that there is a “Goldilocks” zone for indoor RH and that elevated RH can have a negative effect, likely via interaction with PM.

In summary, a wide range of studies support associations between indoor factors such as humidity, wind, and temperature and DE symptoms (Figure 2). Compared to other air pollutants, these data are particularly strong given experimental approaches (environmental chambers [74] and goggles [75,76] to support observational studies [71,72,73].

#### 6.2.2. The Effect of Indoor Humidity and Temperature on Dry Eye Signs

Low indoor humidity and high indoor temperature can negatively impact DE signs, albeit in varied ways. The most robust studies come from controlled environmental chambers. In one study [74], TBUT and tear volume (via phenol red thread test) decreased, and limbal redness increased, after exposure to low RH (TBUT 11.34 ± 0.13 s to 8.31 ± 0.10 s, *p* < 0.05, volume 25.8 ± 1.9 mm pre-CAE to 22.3 ± 2.3 mm, *p* < 0.05, redness 1.6 ± 0.04 to 1.7 ± 0.03, *p* < 0.05). In another study, 10 individuals with mild to moderate DE (symptoms, TBUT < 10 s, Schirmer < 10 mm) and 10 controls were randomly exposed to varying humidity (5%, 40%, and 70%) at a temperature of 72 °F for 25 min on 3 separate days [79]. Tear parameters were assessed 10 min into the exposure on each day. Overall, tear evaporative rates decreased with increasing RH (100.04 ± 13.77 g/m^2^/h increase from 5% to 70% in DE subjects and 73.82 ± 15.47 in controls, *p* = 0.001). Interestingly, TBUT followed a U shaped curve in both groups with a mean of 4.90 ± 1.66 s at 5%, 6.31 ± 2.21 s at 40%, and 5.90 ± 1.91 s at 70% in the DE group and a mean of 17.80 ± 3.91 s at 5%, 20.70 ± 5.88 s at 40%, and 20.00 ± 5.35 s at 70% in the DE group, suggesting an optimal value in the middle at 40% RH, substantiating the epidemiologic observations that also highlighted a middle value for humidity was best [34]. In another chamber study, 19 patients with DE symptoms (OSDI > 12 but staining ≤ 2) and 20 individuals without symptoms (but with low TBUT) were exposed to a 5% RH chamber for 2 h at 23 °C [80]. Measures of TBUT and staining were performed immediately before and after exposure. Both symptomatic and asymptomatic individuals had increased corneal staining (symptoms: 0.68 ± 0.15 to 1.16 ± 0.14, *p* = 0.003; no symptoms: 0.50 ± 0.15 to 1.30 ± 0.19, *p* = 0.0004) and decreased TBUT (symptoms: 2.78 ± 0.56 s to 1.94 ± 0.24 s, *p* = 0.01; no symptoms: 2.81 ± 0.24 s to 2.13 ± 0.19 s, *p* = 0.001) after exposure to low RH. Inflammation measured via matrix metalloproteinase 9 (MMP-9) tear levels also increased after exposure to low RH.

Exposure to low temperature has also been associated with DE signs. In a study of 12 healthy subjects with an OSDI < 12 and an TBUT > 10, temperatures were varied at a constant RH of 40% [81]. Subjects had two visits and were randomly exposed to 25 °C, 20 °C, and 15 °C at one visit and 5 °C and 10 °C at the other. TBUT, tear evaporation rate, lipid layer thickness, and ocular surface temperature were measured at each temperature after 10 min of exposure. Interestingly, mean TBUT levels significantly increased from 7.31 s at 5 °C to 12.35 s at 25 °C (*p* < 0.05), suggesting greater stability at higher temperatures. However, tear evaporation rates increased threefold from 5 °C (0.056 mL/min) to 25 °C (0.17 mL/min), *p* = 0.001 and ocular surface temperature was reduced by ~4 °C, suggesting 25 °C preserved the tear film better than cold temperatures like 5 °C. Median LLT grade also increased with increasing temperature (grade 2 or 20–40 nm at 5 °C and 10 °C and grade 3 or 40–90 nm at 15 °C, 20 °C, and 25 °C). Taken together, these data suggest that ambient temperature has a complicated effect on tears with different tear parameters being adversely affected at colder (stability, LLT, TBUT) and warmer temperatures (TER).

Increasing humidity can improve DE signs, as it was shown to do for DE symptoms. In the desktop humidifier study [77], tear film lipid grade, TBUT, and tear meniscus height were measured after one hour with and without the humidifier. In both environments, tear meniscus height and lipid-layer grade remained constant throughout the hour. However, TBUT increased by a median of 4 s in the humidifier group and TBUT and local humidity level were significantly correlated with one another (Spearman ρ = 0.59, *p* < 0.001). This finding is similar to that of the PM studies where TBUT was the factor most related to air pollution as well. The use of goggles can also improve DE signs. When subjected to wind exposure [76], blink frequency increased from ~19.0 ± 10.0 times/min to ~33.0 ± 10.0 times/min in the group not wearing spectacles and from ~18.0 ± 6.0 times/min to ~29.0 ± 4.0 times/min in individuals wearing conventional spectacles (*p* < 0.05). However, blink rate was unchanged in those wearing goggles. In a similar manner, TBUT decreased after wind exposure from ~5.0 ± 0.5 s to ~2.0 ± 0.5 s in the no spectacle group and from ~5.5 ± 2.5 s to ~5.0 ± 0.5 s in the conventional spectacle group (*p* < 0.05). However, TBUT did not significantly change in the goggle group. Overall, experimental studies show that DE signs can be improved by manipulating temperature [81] and humidity [77,79,80]. Additionally, goggles can blunt the effect of wind exposure on symptoms, blink rate, and TBUT [76]. Overall, these data show that both outdoor and indoor environmental factors can impact DE symptoms and signs (Figure 2). The strongest data come from controlled environmental chambers which highlight how change in temperature and humidity can affect DE symptoms and signs in individuals with and without preexistent ocular conditions.

### 6.3. Potential Pathophysiology

As demonstrated by the studies above, conditions such as wind, humidity, and temperature can directly affect tear evaporation rate and stability. Providing additional support, in a study using mucin, lysozyme, and lactoglobulin to form an artificial tear film, temperature was increased from 24 °C to 34 °C. Evaporation rate measured gravimetrically was 2.97 ± 0.04 μm/min at 24 °C (standard error of the mean, *n* = 41) and 8.0 ± 0.5 μm/min at 34 °C (standard error of the mean, *n* = 5) [82]. Subsequent damage to the ocular surface can trigger inflammatory cascades through mediators like IL-6, MMP-9, and others which can further contribute to DE symptoms and signs [83]. Support for this statement comes from experimental studies in humans. In one study [80], epidermal growth factor (EGF) tear levels decreased significantly (1872.1 ± 340.7 pg/mL to 1107.1 ± 173.6 pg/mL, *p* = 0.007) while IL-6 levels increased significantly (29.6 ± 5.8 pg/mL to 54.3 ± 8.3 pg/mL, *p* < 0.001) after exposure to 5% RH. Levels of MMP-9 also increased significantly after exposure to low RH in individuals with dry eye (10,054.4 ± 7326.6 pg/mL to 25,744.5 ± 13,212.4 pg/mL, *p* = 0.008) and in asymptomatic controls (10,620.5 ± 4494.3 pg/mL to 16,398.7 ± 5538.3 pg/mL, *p* = 0.03).

Similarly, in an air cabin simulation study, inflammatory mediators including epidermal growth factor (EGF), CX3CL1/fractalkine, interferon (IFN)-γ, IL-10, IL-12p70, IL-17A, IL-1β, IL-1RA, IL-2, IL-6, CXCL8/IL-8, CXCL10/IP-10, CCL5/RANTES, TNF-α, and VEGF were collected before and after exposure to 23 °C, 5% relative humidity, localized air flow, and 750 millibars (mb) of barometric pressure. Tear levels of IL-6 and MMP-9 significant increased from 41.2 ± 7.8 to 58.0 ± 7.3 (*p* ≤ 0.05) and from 1881.7 ± 728.3 to 7510.4 ± 3681.8 (*p* ≤ 0.05), respectively, while levels of EGF significantly decreased from 1457.2 ± 249.6 to 1068.7 ± 190.4 (*p* ≤ 0.05) [84].

Beyond inflammation, altitudes greater than 3000 m can also affect the tear film via by hypoxic mechanisms. As altitude increases and barometric pressure falls, the partial pressure of oxygen decreases. A decrease in oxygen can affect the ocular surface though mediators such as delta-like ligand 4 (DII4)/Notch signaling and hypoxia-inducible factor-1 alpha (HIF-1α), which can affect lymphangiogenesis in the lacrimal and other glands [85].

## 7. Mitigation Strategies

Several mitigation strategies can be considered to lower concentrations of air pollutants from indoor and outdoor sources. Exposure management through eliminating sources of pollution, avoiding known sources of pollution, and possibly countering pollution through interventions can reduce both sources of pollutants and manifested disease. While our review separates air pollutants by PM, gaseous pollutants, and weather, when looking at mitigation strategies, it is important to note that some sources may be responsible for multiple pollutants, such as a stove can be responsible for PM and CO. Additionally, mitigation strategies for indoor environments may be beneficial in buildings with poor air quality as the EPA does not regulate indoor levels of pollution as it does for outdoor levels. Moreover, managing outdoor air pollution is typically beyond an individual’s control. Nevertheless, there are targeted interventions to mitigate PM, gaseous pollutants, and adverse environmental conditions indoor and outdoor. While there is crossover of pollutants in the indoor and outdoor environments, sources of emissions can vary, and environmental conditions indoor and outdoor can affect the availability of particulates, for example, as higher humidity increases particle size. Additionally, while there is a general acknowledgement of the environmental component to DE, there additionally have been associations with DE and disease states that are unrelated to pollutants such as Sjogren’s disease, depression, posttraumatic stress disorder [86], and others.

### 7.1. Indoor Mitigation Strategies

Steps to reduce indoor PM levels include replacing filters on central heating and cooling systems, installing particulate air purifiers, and avoiding unvented stoves and fireplaces. While not studied directly in DE, similar environmental interventions have been helpful in mitigating other diseases, such as asthma. In a randomized control trial of 937 children with atopic asthma in the U.S., caretakers in the intervention group were asked to perform mitigation behaviors that were tailored to each child’s skin-test-sensitization results for one year [87]. These included high-efficiency particulate air purifiers, allergen impermeable covers on mattresses and pillows, and specific allergy interventions such as pest control in children with cockroach allergies. In the other group, no interventions were undertaken. Subjects were contacted every two months and asked about the number of days with symptoms such as wheezing, chest tightness, cough, disturbed sleep or decreased play due to asthma in the last two weeks before the phone interview. The group that underwent environmental interventions had fewer symptoms days than the control group (3.39 ± 0.12 days vs. 4.20 ± 0.12 in a 14-day period, *p* < 0.001). Similarly, individualized tailored strategies may be beneficial for individuals with DE symptoms. However, it should be considered that installing indoor interventions such as an air purifier can deteriorate other environmental conditions by increasing noise or light pollution.

Plants can be considered as mitigation strategies for reactive VOCs indoors. For instance, one study assessed the effectiveness of O. microdasys in removing air pollutants [88]. They found that O. microdasys was able to remove 2 ppm of various VOCs from air in the test chambers completely after 48 (benzene), 55 (toluene), 47 (xylene), and 57 (ethylbenzene) hours. Other strategies that can reduce indoor VOCs include avoiding the use of aerosol consumer products such as hairsprays, air fresheners, deodorants, insecticides, and organic cleaning solvents. Additionally, storing VOC containing products in air-tight containers or outside the house and increasing ventilation through open windows, clearing air vents, and installing under-door air vents can reduce indoor VOC levels.

In regard to minimizing the effects of varying temperatures and humidity, indoor humidifiers and goggles can be used by individuals susceptible to environmental stress. Based on the U curve presented above [34] and according to EPA guidelines, optimal indoor relative humidity should be set between 30 and 50%. According to the American Society of Heating, Refrigerating and Air-Conditioning Engineers, indoor temperature should be set between 20–25 °C [89].

### 7.2. Outdoor Mitigation Strategies

While outdoor mitigation strategies are harder to put in place, several strategies have also been suggested to reduce concentrations of gaseous pollutants outdoors. For example, a study that used hourly meteorological and pollution concentration data from across the United States demonstrated that urban trees remove large amounts of air pollution that consequently improve urban air quality [90]. Pollution removal (O_3_, NO_2_, SO_2_, CO) varied among cities with total annual air pollution removal by US urban trees estimated at 711,000 metric tons.

In another study, 213 individuals living in urban areas of Louisville, Kentucky, were profiled for vegetation near their residence, and individual levels of VOC exposure were quantified by measuring 18 urinary metabolite levels of 15 VOCs [91]. An inverse correlation was noted between 8 urinary metabolites of VOCs (acrolein, acrylonitrile, benzene, 1-bromopropane, ethyl benzene, styrene, propylene oxide, and xylene) and vegetation within 100 m of the participant’s residence, indicating that living within 100 m of greenness was associated with lower VOC exposure. Vegetation and exposure mitigation should be considered as mitigation strategies for pollutants both indoor and outdoor VOCs. Outdoor VOCs can be further reduced by minimizing the use of pesticides and controlling and reducing emissions from motor vehicles and industrial and commercial processes. As such, both indoor and outdoor plants appear to be helpful mitigation strategies for reactive gas pollutants.

Ultimately, various air pollutants are present in the workplace, schools, homes, outdoors, and in many of our daily environments. Protection through eliminating, avoiding, and thereby reducing exposures along with implementation of interventions can mitigate the risk of air pollution on human health, particularly on the ocular surface. It is important to note, however, that environmental factors cannot be evaluated in isolation as they impact other environmental measures, such as particulate matter.

## 8. Conclusions

While DE can arise from a multitude of etiologies including concomitant with autoimmune diseases, as a side effect of medications and as a result of aging, this review highlights that environmental conditions can impact DE symptoms and signs, both in individuals with pre-existing DE and in healthy controls. The reviewed studies highlight relationships between PM, bioaerosols, gases pollutants, low and high humidity, high temperatures, and high altitudes with various aspects of DE. However, findings were not always uniform across studies. The most robust studies involved indoor humidity and temperature, which are particularly easy to modify. Overall, addressing environmental conditions can be a promising therapeutic strategy in DE, particularly indoors. The first step of the process is to identify individual level exposures in the workplace and home environment. Subsequently, personalized mitigation strategies can be undertaken to improve air pollution with adding air filters and plants, optimizing temperature and humidity, and removing adverse exposures such as mold, as needed. Future studies are needed to more robustly evaluate relationships between indoor environmental conditions such as lifestyle and living environment on DE symptoms and signs, considering potential confounders and interactions between pollutants and environmental factors. Then, studies can evaluate which environmental manipulations are most beneficial in modifying disease status.
